# Bio-based coatings for reducing water sorption in natural fibre reinforced composites

**DOI:** 10.1038/s41598-017-13859-2

**Published:** 2017-10-17

**Authors:** T. H. Mokhothu, M. J. John

**Affiliations:** 1 CSIR Materials Science and Manufacturing, Polymers and Composites Competence Area, Port Elizabeth, South Africa; 2 Department of Chemistry, Faculty of Science, Nelson Mandela University, Port Elizabeth, South Africa

## Abstract

In this study, bio-based coatings were used for reducing water sorption of composites containing flame retardant treated natural fibres and phenolic resin. Two types of coatings; polyfurfuryl alcohol resin (PFA) and polyurethane (PU) were used on the composites and compared with a water resistant market product. Uncoated and coated samples were conditioned at 90 °C and relative humidity of 90% for three days and the relative moisture content and mechanical properties after conditioning were analysed. In addition, the changes in the weight loss of the conditioned samples were also investigated by thermogravimetric analysis. The moisture diffusion characteristics of coated laminates were also studied at room temperature under water immersion conditions. PFA coated samples showed better moisture resistance and mechanical performance than other bio-based coatings when subjected to long term environmental aging.

## Introduction

The growing interest in using natural fibres as reinforcement in polymeric based composites is mainly due to their abundance, renewable origin, relatively high specific strength and modulus, light-weight, low cost and biodegradability when compared to glass fibres^1^. This is driven by vast demands of new eco-friendly materials that present low environmental impact^2^. In contrast, a serious problem of natural fibres is their strong polar character which creates incompatibility with most polymer matrices as well as compounding difficulties. This leads to non-uniform dispersion of the fibres in the matrix and therefore impairs the properties of the resulting composites. In addition, natural fibres are susceptible to moisture absorption especially when exposed to external factors (temperature and humidity) during their application. These external factors can result to high moisture absorption in the fibres and can cause the deteriorations of the functional, physical, and mechanical properties of the composite materials, due to the formation of micro-cracks on the surface and in the bulk of the material leading to peeling and surface dissolution of the composite^3,4^. This could lead to mechanical failure of the composites and obstruct their use in high-tech industrial applications. Studies on surface modification of natural fibres and its effects on moisture absorption have been reported in literature^5–15^. These studies highlighted (i) the reduction of moisture absorption in natural fibres through chemical treatment with emphases on short term water immersion, (ii) improvement of the interfacial adhesion between the fibres and polymer matrices and (iii) the influence of fibre loading on moisture absorption. However, effects of environmental aging and the development of new strategies to improve moisture resistance of natural fibre composites were not addressed.

A review on hygroscopic aging of cellulose fibres and their biocomposites addressed the effects of environmental aging and new strategies to improve moisture resistance in natural fibre reinforced composites^16^. The review presented (i) the application polyfurfuryl alcohol (PFA) and polyurethane (PU) as possible bio-based coatings to impart moisture resistance in natural fibre reinforced composites; (ii) the application of organic-inorganic material (by pad or dip coating) to induce hydrophobicity or water resistance on fibre surface and (iii) highlighted the importance of investigating the influence of environmental aging on natural fibre reinforced composites. On the application of organic-inorganic material to induce water resistance on fibre surface, most approaches focused on reducing the surface energy in textile substrates by using coating techniques such as sol–gel process, etching, chemical vapour deposition and liquid flame spray, which effectively enhances hydrophobicity. However, the water resistance is mostly studied by water contact angle which is not sufficient for studies on natural fibre reinforced composites. In the case of polyfurfuryl alcohol and polyurethane, most studies reported on the production of water resistance films and protective coatings from PU^17–19^ while PFA was used for the preparation of biocomposites^20–24^. However, incorporation of natural fibres in PFA showed tendencies of moisture absorption due to the hydrophilic nature of natural fibres. For instance, Kumar *et al*.^20^. worked on the development of biofilms and reported a maximum water uptake of 50% for PFA/SPI films due to the presence of water absorbing material, which was soy protein isolate (SPI). In the case of Linganiso *et al*.^24^. the hydrophobicity of PFA decrease with increasing microfiber loading for PFA/agave based composites. This was attributed to the availability of higher number of hydroxyl groups in the composite. In another study, flame retardant treated flax fibre reinforced phenolic composites showed a decrease in the mechanical properties after the composites were exposed to environmental aging^25^. In this case, the decrease was attributed to the moisture absorption of flax fibre and the hygroscopic nature of the applied flame retardant. Most of these approaches had detrimental effects caused by moisture absorption due to the presence of natural fibres. Again there is no much work done on (i) the influence of environmental aging on the mechanical properties of bio-coated natural fibre reinforced composites and (ii) the effects of aging on treated (fire retardant and/or chemical) natural fibre reinforced composites^25,26^. Hence, in our approach, flame retardant treated flax fibre phenolic reinforced composites prepared by compression moulding were coated with polyfurfuryl alcohol and polyurethane as suitable bio-based coatings, and were compared with a water resistant market product. Therefore, this approach does not apply principles related to the coating techniques previously mentioned, but using renewable and sustainable hydrophobic polymer matrices as suitable protective coatings for natural fibre reinforced thermosetting matrix composite. Our understanding is that exposing uncoated and coated samples to environmental conditions such as high relative humidity and temperature can cause changes on the properties of the resulting composites materials. Thus, in this study a comparison between different coatings was applied to overcome detrimental effects caused by moisture absorption in natural fibres composites at high relative humidity and temperatures on the thermal stability, mechanical and morphological properties. The study shows that application of bio-based coatings especially PFA can significantly reduce moisture absorption of fibre reinforced composites and uphold their structural integrity when exposed to environmental conditions.

## Preparation Methods

### Materials

Furfuryl alcohol (FA) was donated by Illovo Sugar Limited based in Umhlanga, Durban, South Africa. *P-*toluenesulfonic acid monohydrate catalyst were procured from Sigma-Aldrich, South Africa. Aliphatic polyether polyurethane (PU) without free isocyanate groups was a gift sample from Alberdingk Boley, Germany. Water resistant market product (FIRESHELL^®^ (F1E)) was a gift sample from TPR^2^ Corporation, USA. FR Flammentin TL833 flame retardant (FR) with a decomposition temperature of 155 °C was supplied by ACTI-Chem, South Africa. The major component of the Flammentin TL833 is diammonium phosphate (DAP).

### Preparation of flax/phenolic composites

Woven flax fabrics (47 cm × 33 cm) were subjected to flame retardant (FR) treatment by using a padder (Roaches BVHP). The flame retardant was prepared and then padded onto the fabric followed by drying at 120 °C for 1 minute. The dry loading of FR on fabric is approximately 32–35%. Flame retardant treated woven flax fabric (12 layers) was impregnated with the Eponol resin 2485 and pre-pregs were prepared by heating in an oven at 120 °C for 10 minutes. Laminates were prepared by compression moulding the pre-pregs at 135 °C for 80 minutes.

### Bio-based coating of flax/phenolic composites

**Figure 1:**
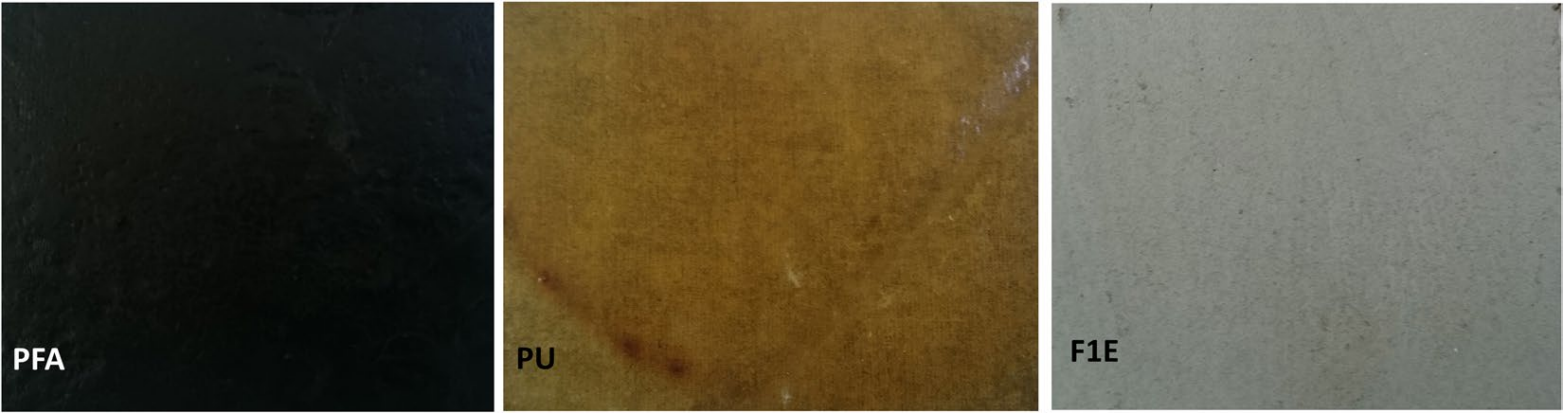
Flax/Phenolic laminates coated with polyfurfuryl alcohol (PFA), polyurethane (PU) and FIRESHELL^®^ (F1E).

Woven flax fibre reinforced phenolic laminates were each coated with three different bio-based coatings namely: water resistant market product (FIRESHELL® (F1E)), polyurethane (PU) and poly(furfuryl alcohol) (PFA) which was polymerized from furfuryl alcohol (FA) (Fig. 1). The polymerization of furfuryl alcohol was achieved by use of a catalytic solution of p- toluenesulfonic acid (0.3 g/100 ml); the mixture was kept in a freezer for 5 days till the viscosity was stable enough for coating. The coated laminates were left to dry until the surfaces were completely dry.

## Characterization

### Water absorption

The coated samples were tested for water absorption properties according to ASTM D570 for 7 days. Three samples from each coated laminate were dried at 50 °C under vacuum for 24 hours. The samples were then cooled to room temperature in a desiccator, weighed to the nearest 0.001 g immediately, and immersed in distilled water at room temperature. Weight measurements were done after wiping the samples with paper towel to remove excess surface water. The percentage of water absorption (*W*_*a*_) was calculated based on the gained weight after immersion compared to the dried sample weight determined according to Equation 1:

1 $${W}_{a}=(\frac{{W}_{t}-{W}_{o}}{{W}_{o}})\times 100 \% $$

where *W*_*t*_ is the weight of the sample at time t and *W*_*o*_ is the initial weight of the sample.

### Environmental aging

The coated samples were aged in a Binder GmbH environmental chamber model APT.line^TM^ KMF (E5.2) set at 90 °C and 90% RH for 3 days. Three samples from each coated laminate were weighed before and after aging according to ASTM D5229. The percentage of relative moisture content was calculated based on the gained weight after aging according to Equation 1.

### Thermogravimetric analysis

The thermal properties of coated and uncoated flax/phenolic composites were carried out on a PerkinElmer Pyris 1 thermogravimetric analyser (TGA). Environmetally aged samples with masses in the range of 5 to 10 mg were heated from 30 °C to 700 °C under a nitrogen flow of 20 mL min^−1^ at a heating rate of 10 °C min^−1^.

### Tensile properties

Tensile testing of the aged samples was carried out using an Instron tensile tester in accordance with ASTM D638. Samples of dimensions 125 × 10 × 4 mm were tested at a speed of 10 mm/min and a gauge length of 50 mm.

### Scanning electron microscopy (SEM)

The morphology of environmentally aged tensile samples was examined by a VEGA TESCAN scanning electron microscopy (SEM) from Rhodes University. Cross-sections of the samples were coated with gold by an electro-deposition method to impart electrical conductivity before the SEM micrographs were recorded.

## Results and Discussion

### Water absorption

**Figure 2:**
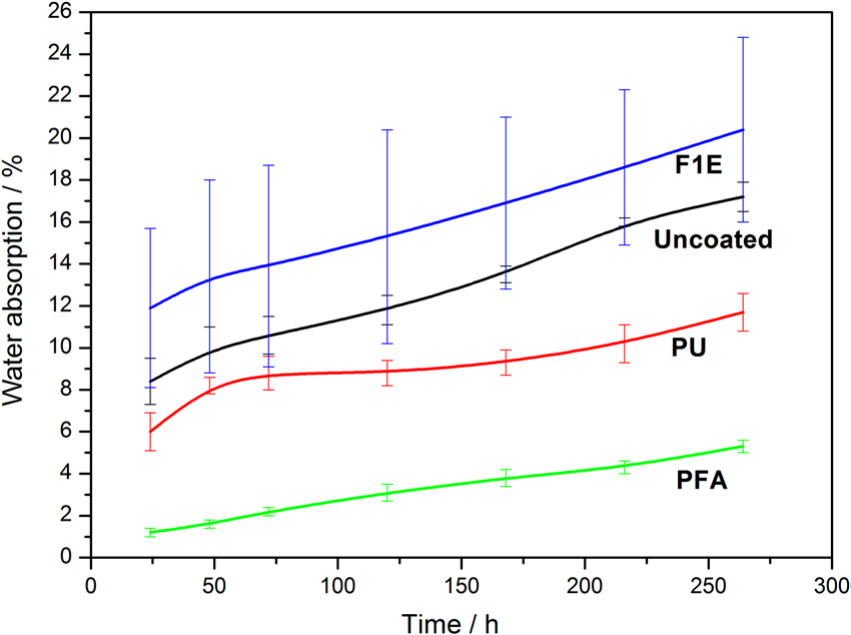
Water absorption behaviour of flax/phenolic laminates coated with different bio-based coatings.

Figure 2 presents the percentage of water absorption results for coated and uncoated flax/phenolic laminates. The water absorption percentage was observed to increase with increasing immersion time for all samples. However, a significant increase in water absorption was observed for F1E coated and uncoated flax/phenolic laminates with increasing time, this was due to their susceptibility to water uptake (Fig. 2). It well-known that commercial fire retardants can be hygroscopic and cause high moisture absorption in treated composites and natural fibres^27^. On the other hand, PU and PFA coated flax/phenolic laminates exhibited reduced moisture absorption compared to F1E and uncoated samples. The reduced water uptake for PU coated laminate is due to good moisture resistance properties possessed by polyurethanes. In polyurethane, the moisture resistance is typically associated with its crosslinking density. For that reason, high crosslinking density is associated to high hydrophobicity^28,29^. For instance, Zhang *et al*.^30^. investigated the properties of polyurethane coatings derived from a series polyester polyols (1,4-cyclohexanedimethanol (1,4-CHDM), trimethylol propane (TMP) and 1,6-hexanediol (HDO)) crosslinked with hexamethylene diisocynate (HDI). The authors reported that the water absorption decreased with increasing molar ratios of TMP or 1,4-CHDM which implied that the PU coatings were properly crosslinked, enabling them to reduce water uptake.

PFA coated laminate attained 75% reduction in water absorption with respect to uncoated and F1E coated laminates. This is attributed to the hydrophobic nature of PFA as reported by Kumar *et al*.^20^. Who studied the development of biofilms from protein isolate and polyfurfuryl alcohol and Dong *et al*.^31^. Who studied wood/polymer nanocomposites. In the case of biofilms, the water uptake of SPFA films immersed for 24 h was found to have decreased by 51%. In the case of wood/polymer nanocomposites, water uptake was reduced by about 60% for FA and FA/SiO_2_ treated wood composite compared to untreated wood composite. In literature, it is well documented that poly(furfuryl alcohol) is polymerized from the reduction of hydrophilic furfural via a cationic condensation reaction in the presence of an acidic catalyst^31,32^. Therefore, it is possible that the number of OH groups in FA available for hydrogen bonding with water molecules were reduced during PFA polymerization, hence resulting in a non-polar polymer.

### Environmental aging

**Figure 3:**
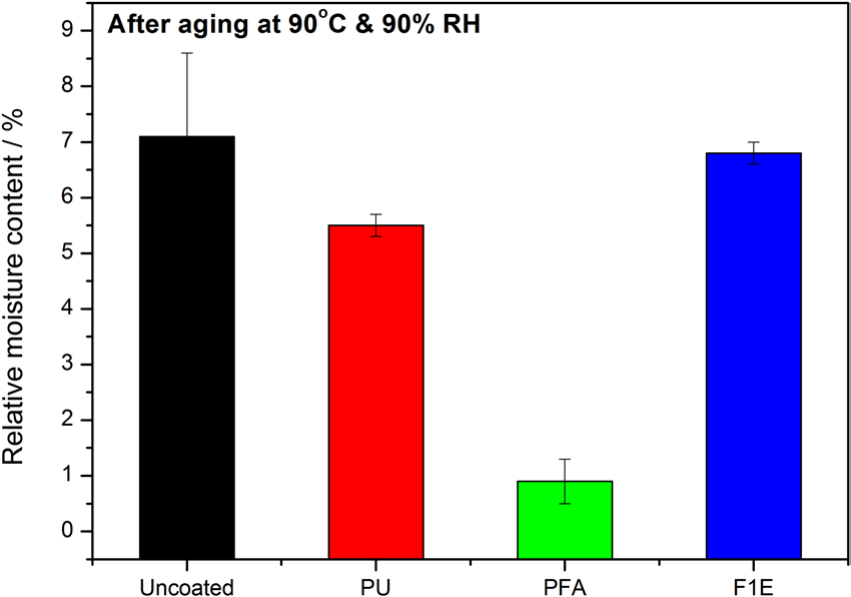
Relative moisture content of uncoated and coated flax/phenolic laminates after aging.

Figure 3 shows the percentage relative moisture content obtained from environmental aging tests. Similarly to the water absorption results, an increase in the relative moisture percentage was observed for uncoated flax/phenolic composite and for both PU and F1E coated laminates due to their susceptibility to high relative humidity (RH) and temperature (Fig. 3). In the case of uncoated flax/phenolic composites and F1E coated laminate, the higher moisture absorption is due to the hygroscopic nature of commercial fire retardants applied on the flax fibre and the flax/phenolic composite respectively. Similarly observation were explained by Molaba *et al*.^25^. On the investigation of ageing and thermal studies of flame retardant treated flax fibre reinforced phenolic composites. The authors reported a prominent decrease in strength of flame retardant composites as a result of the hygroscopic nature of commercial fire retardants. This was attributed to the dissociation of DAP in the flame retardant to form ammonia and phosphoric acid when exposed to high temperatures and humidity^25,26^. On the other hand, PFA coated sample exhibited 1% relative moisture content which implies that the sample was not highly affected by being exposed to high temperature and RH conditions. This is due to the hydrophobic and temperature resistant properties of PFA which reduced the water uptake in the composites and the results are in line with water absorption results. It was reported in the study of biofilms that the hydrophobicity of PFA improved with increase in curing time for PFA-α-SPI films conditioned at 65 ± 2% RH for 3 days^20^.

### Thermogravimetric analysis

**Figure 4:**
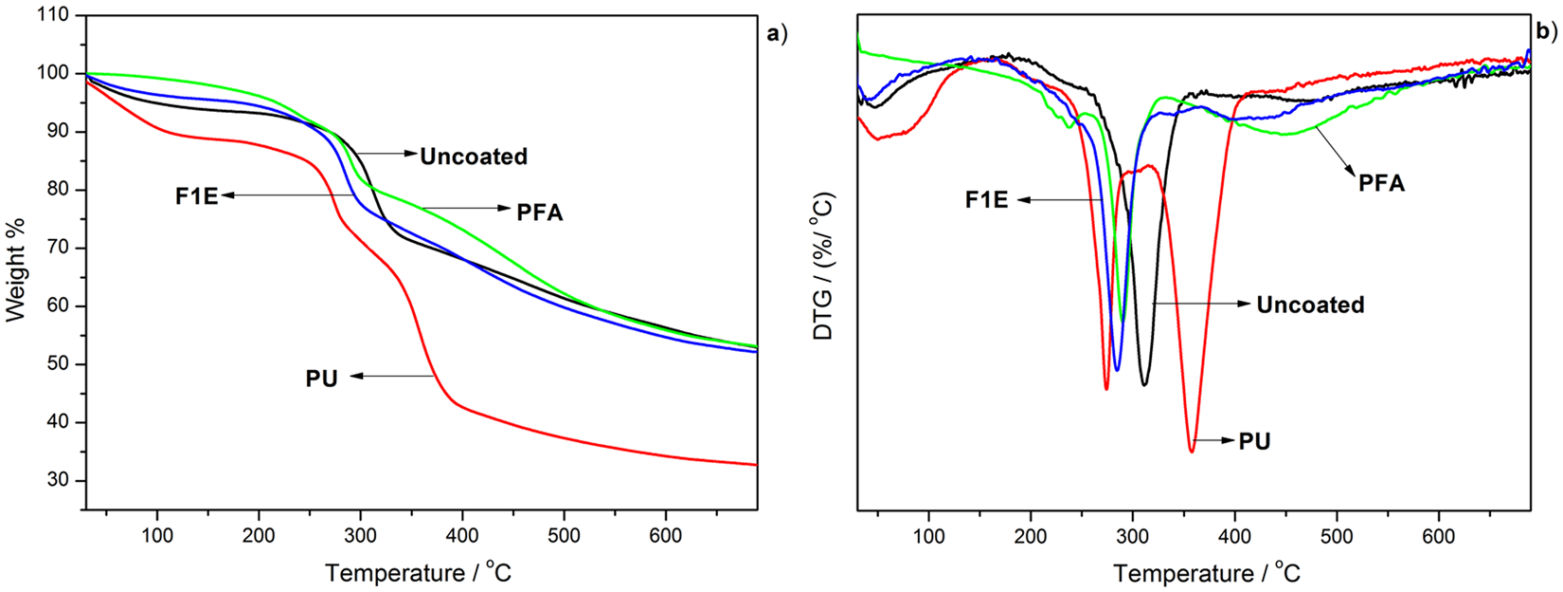
(**a**) TGA and (**b**) DTG curves of phenolic/flax fibre laminates after environmental aging.

The thermograms of coated and uncoated flax/phenolic composites are shown in Fig. 4. Thermogravimetric analysis (TGA) was used to investigate the effect of environmental aging on the weight loss of bio-based coated flax/phenolic composites. The thermograms of uncoated flax/phenolic composite below 200 °C, showed a weight loss around 5% which was attributed to high moisture absorption. It has been reported that flax fibre reinforced composites tend to absorb rather large amounts of water when exposed to moisture^32^. Similar decreases in the weight loss was observed for both PU and F1E coated laminates as a result of absorbed moisture. On the other hand, PFA showed weight loss around 200 °C attributed to the formation of alkylfurans (e.g., 2-methyl furan and 2-furfuryl-5-methylfuran) due to scission of both methylene and methyne links in PFA^33^. The result showed that environmental aging had little or no influence on the thermal stability of PFA coated composite. The high char residue (≈50–55%) around 600 °C can be attributed to the flame retardant applied on the fibres, PFA and F1E coatings. Therefore it can be deduced that the application of the flame retardant on flax fabric (uncoated sample) delayed the formation of volatile pyrolysis products and the promotion of char formation when the composites are subjected to heat and likewise with PFA and F1E which are characterized as thermally stable coatings. In the case of PU char residue, the absorbed moisture may have reacted with flame retardant resulting in a catalytic effect, thereby reducing thermal stability and char content. From the overall results, the PFA coated laminate showed better thermal stability compared to other coatings after environmental aging.

### Tensile properties

**Figure 5:**
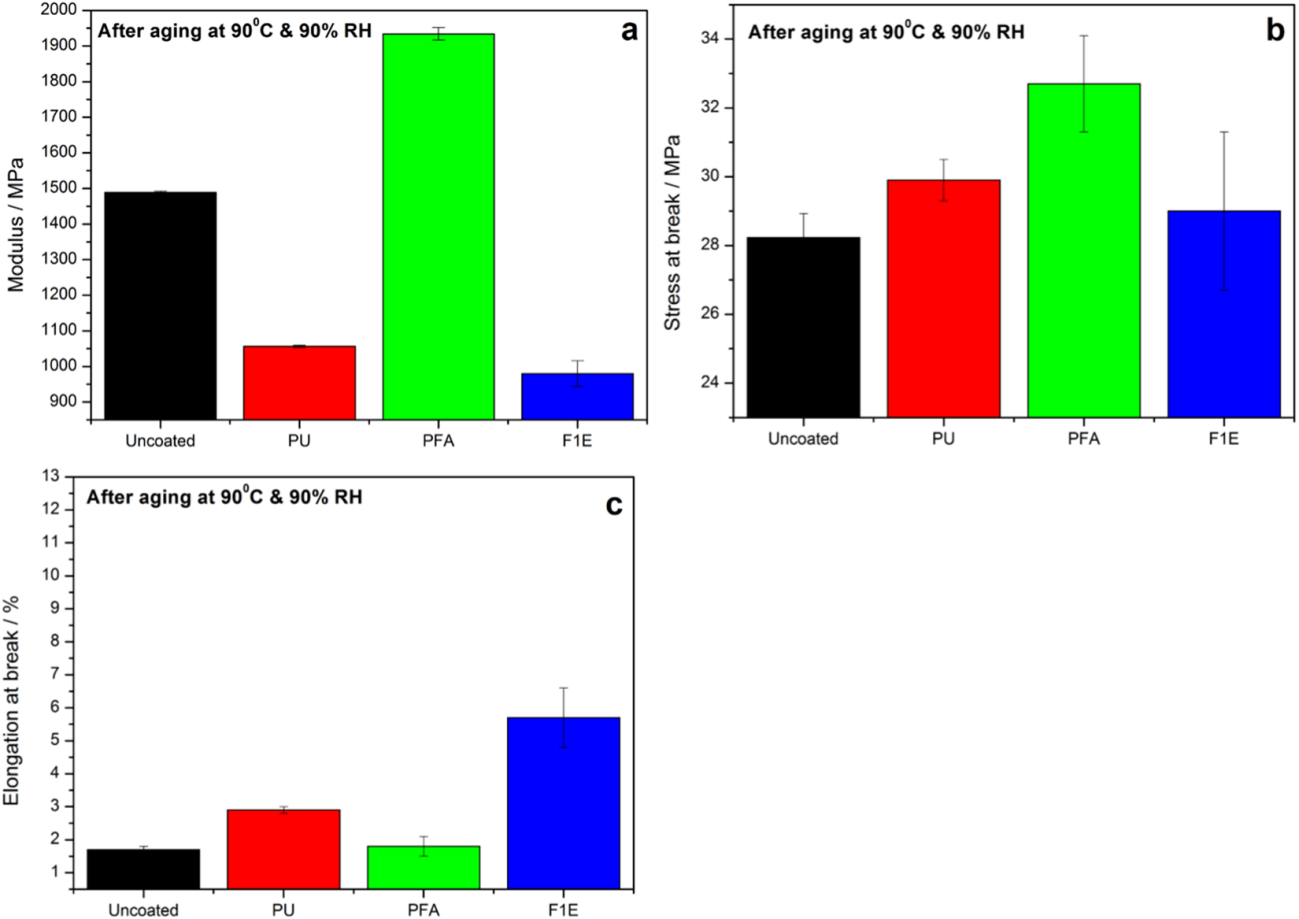
(**a**) Tensile modulus (**b**) Tensile stress at break and (**c**) elongation at break of uncoated and coated phenolic/flax fibre laminates after aging.

The mechanical properties of coated and uncoated flax/phenolic laminates after aging are shown in Fig. 5. It is well known in fibre reinforced composites that penetration of water through the polymer can lead to a disruption of the fibre/polymer interface and result in the reduction of the overall strength of the composite. From the tensile results, it was observed that PFA coated laminate exhibited highest modulus value (1.93 GPa) after being subjected to environmental conditioning compared to uncoated (1.59 GPa); PU (1.05 GPa) and F1E (0.98 GPa) coated flax/phenolic composites (Fig. 5a). This can be attributed to (i) the hydrophobic nature of poly(furfuryl alcohol) which resists the penetration of water within the laminate and (ii) can be possibly be a result of increased brittleness related to changes in phenolic matrix^26^. In the development of biofilms from protein isolate and polyfurfuryl alcohol^20^, the tensile strength and modulus of PFA-α-SPI films conditioned at 65 ± 2% RH for 3 days increased from 7.5 to 20.6 MPa and 30 to 420 MPa respectively compared to neat SPI with tensile strength of 5.8 MPa and modulus of 15 MPa. In addition the moisture content of the conditioned PFA-α-SPI films decreased with increase in PFA curing time and this was attributed to the hydrophobic nature of PFA. Similar observation were also reported in the investigation of kenaf fibre/PFA composites^19^ and on wood/polymer nanocomposites^31^. The authors reported better retention of their mechanical properties after aging as a result of the moisture resistance property of PFA. The PFA and PU coated samples exhibited high stress at break with decreasing elongation at break as a result of undamaged fibre/matrix interface after aging, thereby maintaining the strength and stiffness of the laminates as compared to F1E coated sample (Fig. 4b and c).

In the case of FR, it seems after aging, the composite attains a plasticizing effect due to absorbed moisture. The plasticizing effect is due to (i) hygroscopic nature of commercial flame retardants and (ii) moisture diffusion into thermoset composites generally leads to hydrolysis and plasticization of the matrix. Exposure to environmental aging cause reduction in the interfacial stress transmissibility due to matrix plasticization consequently decreasing the strength, stiffness and modulus of the composite. Moisture absorption is known to cause structural deformation to the composite as a result of absorbed moisture and this can be further observed from the SEM results to be discussed. The diffusion of water in the composite creates hydrogen bonds with the fibre which can lead to the disruption of the interface between the fibre and matrix especially at elevated temperatures and humid conditions. Several studies have reported on the effect of absorbed moisture which causes plasticization and as a result may alter stress transfer and degrade the fibre/matrix interface^34–36^. The studies summarized that the plasticization process by moisture involves the disruption of the Van der Waals’ bonds between hydroxyl groups and as a result the interfacial adhesion between the matrix and fibres becomes weak.

### Scanning electron microscopy (SEM)

**Figure 6:**
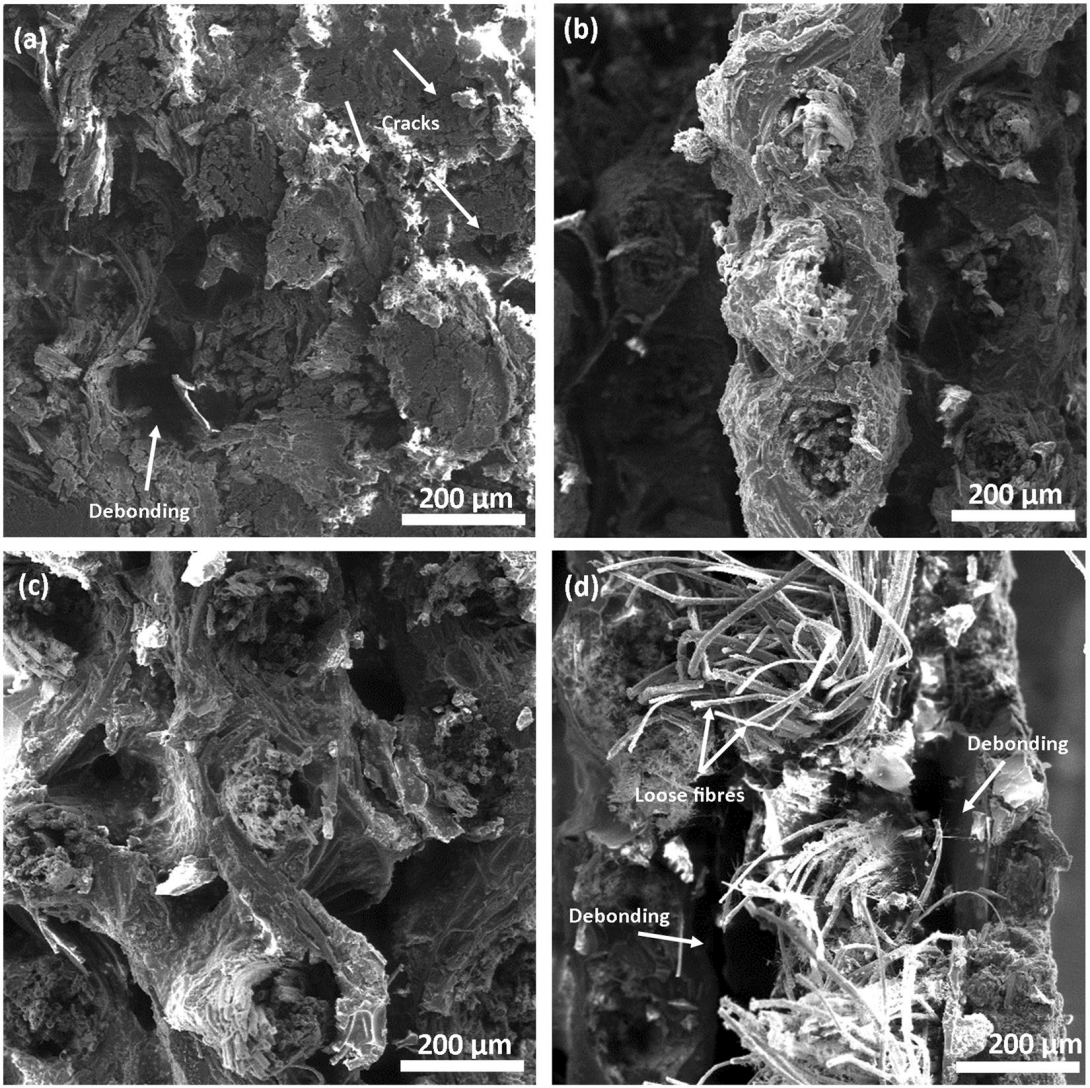
SEM images of flax/phenolic laminates (**a**) uncoated, (**b**) PU, (**c**) PFA and (**d**) F1E.

The SEM images were used to investigate the effects of high relative humidity and temperature on the mechanical properties of uncoated and coated flax/phenolic laminates as shown in Fig. 6. It is well known that moisture absorption in natural fibre reinforced composites causes ‘loss of mechanical properties as a result of structural deformation. The adsorbed moisture causes swelling of the natural fibres, resulting in the formation of micro- cracks and debonding in the matrix as observed from Fig. 6a and d (indicated by arrows). Uncoated and F1E laminates shows deformation of the composites as a result of absorbed moisture during environmental aging at high relative humidity and temperature (Fig. 6a). In the case of uncoated laminate, micro-cracks are observed between the flax fibres and the phenolic resin while F1E coated laminate exhibited significant deformation. Debonding of fibres from the matrix was also observed as well as fibres protruding from the matrix (Fig. 6d, indicated by arrows). The structural deformation of these laminates could further explain the decrease in the tensile properties of flax/phenolic laminates as observed from the tensile results. The flow of water molecules through cracks resulted in the deformation of the fibre/matrix interface which led to the composite failure^16,37^. For PU and PFA coated laminates, no cracks or structural deformation was observed from the composites (Fig. 6b and c). The coatings were able to reduce penetration of water molecules preventing the formation of micro-cracks which could have resulted in weakening of the fibre/matrix interface and caused damages to the composite. The mechanical properties of PFA were better than the PU coated and both uncoated and F1E coated laminates as seen from tensile properties.

## Conclusions

In this work, bio-based coatings were used as possible coatings to reduce moisture absorption as a result of environmental aging in flame retardant treated natural fibre phenolic reinforced composites. The moisture absorption and mechanical performance of bio-based polyurethane and poly(furfuryl alcohol) coated flax/phenolic laminates were compared with laminates coated with a water resistance market coating and uncoated laminate. The moisture absorption properties of PFA coated samples were significantly lower than that of both PU and F1E. Furthermore, PFA coated composites showed a significant reduction in moisture absorption after aging at high temperatures and humidity conditions because of the hydrophobic nature of PFA. The SEM image of PFA coated composite did not show debonding of the fibre/matrix interface after environmental aging at high humidity and temperatures. Hence, PFA attained better retention of mechanical properties when compared to uncoated, PU and F1E laminates after environmental conditioning. Furthermore, the thermal degradation studies showed that the absorbed moisture had little or no influence on the thermal stability of PFA coated composite. The results suggest that PFA can serve as a suitable protective bio-based coating for fire retardant treated natural fibre phenolic reinforced composites with high structural integrity and appreciable mechanical and thermal properties.
